# Assessment of physical activity among adolescents: a guide to the literature

**DOI:** 10.3389/fpsyg.2023.1232382

**Published:** 2023-07-07

**Authors:** Marek Sedlačík, Veronika Lacinová, Kamila Hasilová

**Affiliations:** Department of Quantitative Methods, University of Defence, Brno, Czechia

**Keywords:** physical activity, fitness tests, adolescents, military, state defense

## Abstract

**Purpose:**

The aim of this article is to systematically review articles and annual reports concerning young peoples' physical activity (PA) and linking this to considerations of the state and national defense.

**Method:**

A systematic search of the literature included an analysis of publications accessible in global databases and other available books, student papers, and projects. The articles and reports were categorized based on year of publication, methods used, age of respondents, sample size, country, and digital object identifier (DOI).

**Results:**

The result of this search is an overview of the extent and manner in which the worldwide scientific community is addressing the current situation and the long-term development of the physical fitness of adolescents. This publication also maps to what extent professional publications and articles are addressing PA from the perspective of the needs of armies and armed forces of various countries around the world.

**Conclusions:**

The article provides a systematic overview of methods used to measure PA, and an overview of articles dealing with assessing PA. The examined articles indicate that from the perspective of not only national defense, but also health and overall quality of life, in particular, we need initiatives to encourage and motivate young people to increase their everyday PA. The research therefore also includes an overview of factors that may considerably influence PA. The results ascertained in this publication will be used, *i.a*. for investigating a longitudinal defense research project of the Ministry of Defense of the Czech Republic in which the authors are participating.

## Introduction

This article aims to systematically map whether the global scientific community is addressing the current situation and long-term development of physical fitness in the adolescent population, and if it is, then to what extent and in what manner. This article also aims to check whether such professional publications and articles are looking at physical activity (PA) from the perspective of the armed forces of countries around the world.

One reason for this publication is that the team of authors is involved in the project BODY—Physical Fitness of the Population as a Risk Factor for Ensuring the Defensive Capacity of the Czech Republic (*TELO–Tělesná zpusobilost populace jako rizikový faktor pro zabezpečen*í *obranyschopnosti Ceské republiky*). This is a defense research project of the Ministry of Defense of the Czech Republic in the programme: Development of the Armed Forces of the Czech Republic, main objective: Personnel Preparation (project OYUOB20200001, 2021).

The project aims at designing certified methodologies for selecting and training individuals to deal with physical and mental stress according to the standards of the Czech Armed Forces. These methodologies will be based on a study of the actual physical fitness of students of secondary and vocational schools. This study will include proposals for changing educational and training programmes dealing with developing physical fitness, and to further develop unified approaches to testing and monitoring physical fitness in connection with Ministry of Defense (especially the Czech Armed Forces) requirements. In addition, a functional database will be built enabling the long-term tracking and evaluation of selected criteria and descriptors of physical fitness.

In these COVID or post-COVID times, the project and its expected results are all the more valuable, as the project also has features of a longitudinal study, mapping the development of physical fitness after the resumption of face-to-face learning.

For the reasons stated above, a systematic search and analysis of available publications was out, drawing upon not only professional articles and publications contained in worldwide databases, but also those absent from such sources, namely books, student papers and projects published at the national level.

## Methods

### Strategy for the Literature search

We conducted a search within the electronic databases Scopus, Web of Science, HBSC (Health Behavior in School-Aged Children), and GHO (Global Health Observatory). Search terms included the key words: “physical,” “fitness,” “adolescents,” “measuring,” “military,” and “cadets,” and the terms were combined in groups of three or four with Boolean operator “and.” The year range was first set to 2016–2021. However, several papers from earlier years were added to which the search was led by references in articles. In addition, on the national level we conducted an analysis of available publications and articles, books, student papers, and projects archived in the Moravian Library in Brno since 2006. The search was carried out by all authors with the assistance of a librarian.

### Search outcome and eligibility criteria

The literature search was conducted in two phases. The first consisted of screening the titles and abstracts of records obtained from the databases. There were almost 6,000 articles produced from the initial search phase, i.e., after a search within the databases and library using the key words mentioned above. The primary screening of the titles and abstracts within the databases narrowed down the number of relevant records to approximately 8% of the initial number of articles.

In the second phase of the search, the full texts of the articles were assessed for eligibility. Papers and studies were excluded if they

- concentrated only on specific sports activities (such as basketball, football, etc.) and their influence on PA,
- focused only on psychological aspects connected to physical fitness,
- examined solely medical issues (e.g., the impact of surgery, effects of specific disorders),
- did not have a full text available, and
- were published as a full text in languages other than English or Czech.

**Figure 1:**
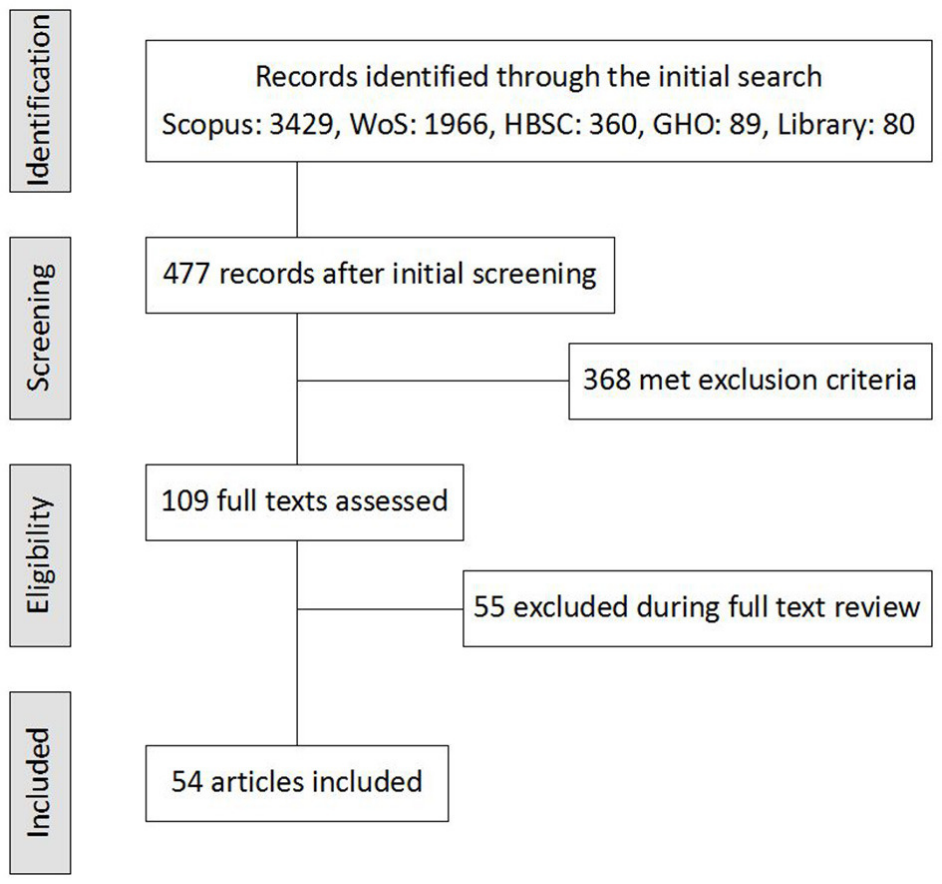
The literature search flowchart.

After removing ineligible articles, the authors selected 109 of the rest for more in-depth study. From this set of articles, 55 papers were excluded because they did not concentrate on PA in connection with its measurement (either using tests or questionnaires), or they connected PA with cognitive processes, or they compared the influence of different organized PAs, or else they did not have a digital object identifier. Finally, 54 articles were included into the systematic review. The overall search strategy is summarized by a flowchart diagram (Figure 1).

Data were retrieved and recorded from the full texts of the included articles: namely the title, authors, and year of publication, and also data describing age, sample size, methods, and country.

## Results and discussion

### Overview of the published studies

PA is one of the main factors influencing one's health, psyche, and overall quality of life. Yet, according to World Health Organization (WHO) estimates, 81% of individuals aged 11–17 years are physically inactive, as they carry out moderate- to high-intensity PA for < 60 min per day (World Health Organization, 2014). In recent years several organizations, expert associations and initiatives have emerged in the field of public health with the main objective of drawing attention to problems associated with physical inactivity. One example is Health Behavior in School-aged Children (International Coordinating Centre, 2021), which is an international alliance (now including 50 countries and regions across Europe and North America) addressing the lifestyles of adolescents aged 11–15 years. This alliance connects scientists from all over the world to cooperate in a cross-national survey of school students. Another noteworthy project is the international project IPEN ADOLESCENT (International Physical Activity and the Environment Network, 2021) sponsored by the United States' National Institutes of Health and coordinated by the research group International Physical Activity and the Environment Network. The project is primarily focused on understanding the relations between the built-up environment, objectively monitored PA, sedentary behavior, and obesity in adolescents.

**Figure 2:**
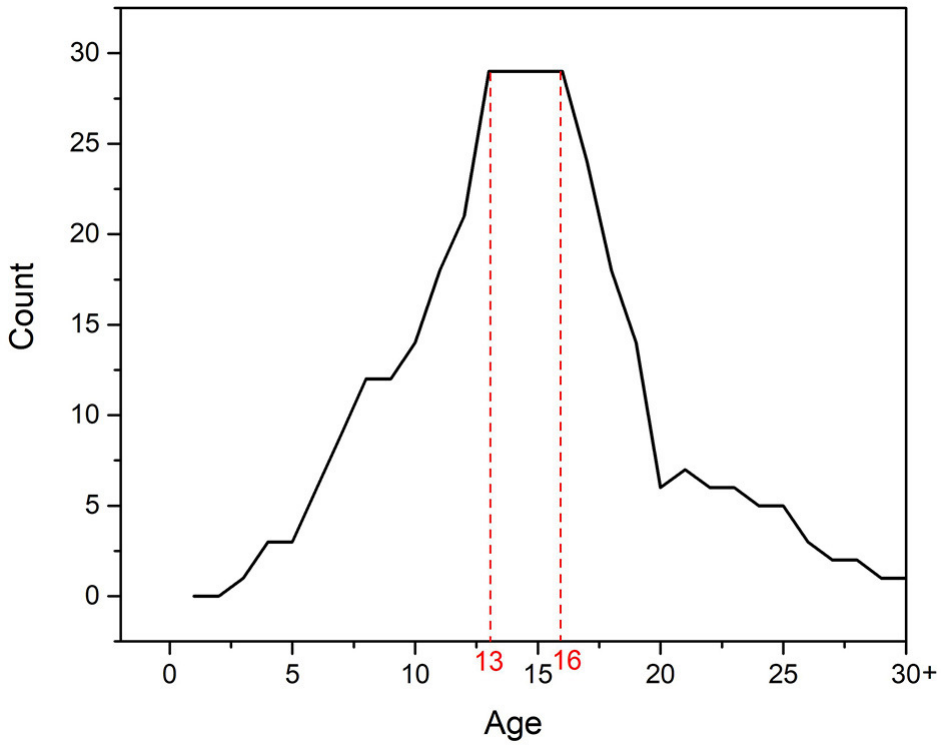
Number of relevant articles with regard to the age of respondents.

Data from our literature search cover the age range from 3 to 28 years. Only one examined publication also included older respondents. Most articles included in the search related to adolescents aged 13–16 years (Figure 2).

### Methods of measuring PA

**Figure 3:**
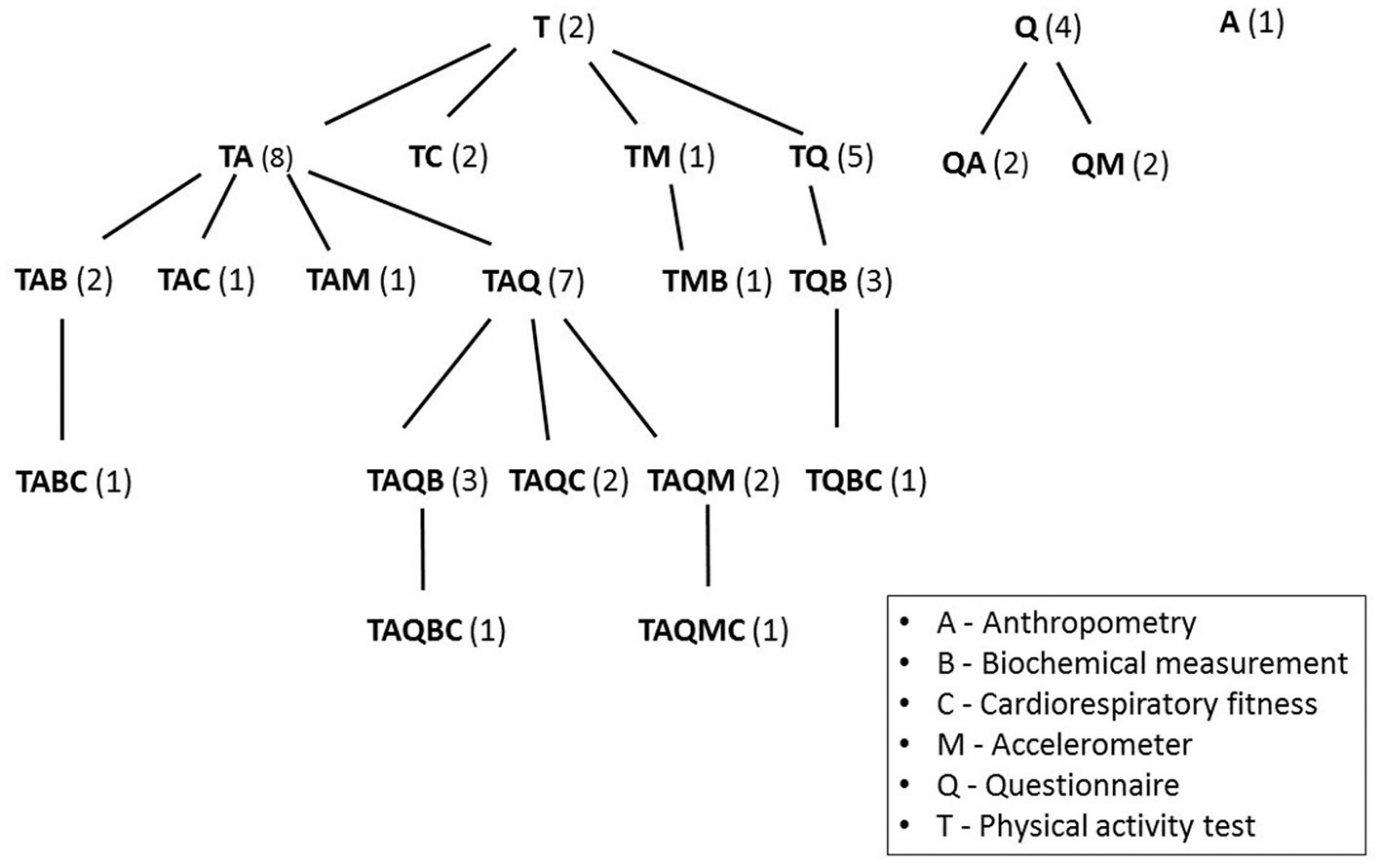
Methods (and combinations of methods) for measuring fitness and PA.

To date, a wide range of methods has been used to measure PA in children and adolescents. These include self-reporting methods such as questionnaires, activity logs, and diaries, as well as objective measures of PA such as direct observation, heart rate monitoring, accelerometers, and pedometers. Certain selected methods for measuring fitness and PA (or combinations of such methods) are shown in Figure 3. Specifically, these methods are: PA test (T), anthropometry (A), cardiorespiratory fitness (C), accelerometer measurement (M), biochemical analysis (B), and a questionnaire survey (Q). The number in brackets shows the number of articles included in the search that used such a combination of methods and corresponds to the column “Method” in Table 1. The combination of a PA test and anthropometry was used most often. This combination supplemented with a questionnaire was also common. To get an accurate picture of fitness, it is appropriate to supplement a questionnaire survey with a PA test, because some studies point out that respondents tend to overestimate the time spent doing PA, and when estimating time spent doing moderate to high intensity PA, also include low-frequency activities (Wallston et al., 1978). Overestimating one's PA may also result from the broad spectrum of PAs given (Fogelholm et al., 2006).

**Table 1:** List of articles concerning the assessment of physical fitness of adolescents; “L” written beside the year means that it was a longitudinal study, and “m” written beside the country means that the study was focused on soldiers (or conscripts) in the given country.

**Year**	**Method**	**Age**	**Sample size**	**Country^a^**	**DOI**
**2021**	T, Q	11–13	971	FI	10.1111/sms.13847 (Joensuu et al., 2021)
**2021**	T, Q, B	13	795	IE	10.1177/1356336X20917416 (O'Keeffe et al., 2021)
**2020**	Q	13–19	1,340	PL	10.3389/fpubh.2020.00466 (Kuśnierz et al., 2020)
**2020 L**	T, C	3–17/ 4–23	3,742	DE	10.3389/fpubh.2020.00458 (Niessner et al., 2020)
**2020 L**	T, Q, B	13–14/ 16–17	93	BR	10.1016/j.jesf.2020.06.002 (Roldão da Silva et al., 2020)
**2020**	Q,	12–17	128	US m	10.3390/ijerph17010024 (Pearlman et al., 2019)
**2020**	Q, A	19–26	1,243	LT m	10.3390/ijerph17030783 (Mieziene et al., 2020)
**2020**	T, A, Q	15–18	1,036	HR	10.13112/PC.2020.4 (Kasović et al., 2020)
**2020**	T, M	19	34	FI m	10.1249/MSS.0000000000002092 (Jurvelin et al., 2020)
**2019**	T, Q, B	21	537	US	10.18666/TPE-2019-V76-I1-8462 (Burner, 2019)
**2019**	T, A, Q	18–26	186	QA	10.1371/journal.pone.0223359 (Chrismas et al., 2019)
**2019**	T, A	7–18	17,618	CH	10.1371/journal.pone.0220863 (Lang et al., 2019)
**2019**	T, Q, B, C	6–17	3,800	CA	10.24095/hpcdp.39.3.02 (Arena et al., 2019)
**2019**	T, A, Q	15–17	1,036	HR	10.1371/journal.pone.0219217 (Štefan et al., 2019)
**2019**	T, A, Q	11–17	99	US	10.7759/cureus.6262 (Arena et al., 2019)
**2019**	T, A, B, C	7–8	1,246	CH	10.1016/j.rmed.2019.105813 (Köchli et al., 2019)
**2019**	T, A	6–18	560	AT	10.3390/ijerph16214117 (Ruedl et al., 2019)
**2019**	T, A, Q, M	14–16	233	ES	10.1016/j.scispo.2018.10.014 (Corredor-Corredor et al., 2019)
**2019**	T, A	8–10	80	GB	10.1123/pes.2018-0135 (Weston et al., 2019)
**2019 L**	T, A, Q, C	18–19	1,008,787	SE m	10.1016/j.jadohealth.2019.02.016 (Lissner et al., 2019)
**2019**	T, A, C	13–16	413	EE	10.3390/ijerph16224479 (Galan-Lopez et al., 2019)
**2018**	T, A, Q, C	8–11	122	AU	10.1123/jmld.2016-0076 (Barnett et al., 2018)
**2018**	T, A, Q, M, C	11–13	576	GB	10.1007/s11136-018-1915-3 (Eddolls et al., 2018)
**2018**	T, Q	13–15	382,259	TW	10.1038/s41598-018-34370-2 (Hsieh et al., 2018)
**2018**	T, A	17–18	28	RU m	10.7752/jpes.2018.s5300 (Kudryavtsev et al., 2018)
**2018**	T, C	11	265	US	10.1186/s12889-018-5107-4 (Hsieh et al., 2018)
**2018**	T, A, Q, B, C	12–18	734	PT	10.1007/s00431-018-3164-4 (Agostinis-Sobrinho et al., 2018)
**2018**	Q	15–17	1,846	CZ,PL	10.21101/cejph.a4521 (Frömel et al., 2018)
**2018 L**	Q, M	14 (19)	116	NO	10.3389/fpubh.2018.00097 (Lagestad et al., 2018)
**2018**	T, A, Q	6–18	335,810	GR	10.3389/fnut.2018.00010 (Arnaoutis et al., 2018)
**2018**	Q	12–13	749	US	10.1016/j.amepre.2018.01.030 (Nicosia and Datar, 2018)
**2017**	T	14–17	143	ES	10.1007/s00431-016-2809-4 (Fernández et al., 2017)
**2017**	T, A, M	10–17	2,698	PT	10.1186/s12966-017-0481-3 (Júdice et al., 2017)
**2017**	T, A, Q, B	13–16	444	E6^b^	10.1016/j.jsams.2016.08.003 (Cadenas-Sanchez et al., 2017)
**2017**	T, M, B	7–12	365	CZ	10.1186/s12199-017-0629-4 (Gába et al., 2017)
**2017**	Q, A	10–19	3,337	IN	10.7860/JCDR/2017/27080.10870 (Dave et al., 2017)
**2017**	T, A	12–13	31	KR	10.12965/jer.1735132.566 (Cho and Kim, 2017)
**2017**	T, A	14–15	354	RS	10.1186/s12889-017-4727-4 (Tishukaj et al., 2017)
**2017**	T, A	13–18	14,794	KR	10.7570/jomes.2017.26.1.61 (Lee et al., 2017)
**2016**	T, A, Q, M	18+	1,607	E7^b^	10.1371/journal.pone.0150902 (Marsaux et al., 2016)
**2016**	T, A, B	14–16	357	US	10.18666/TPE-2016-V73-I1-5903 (Davis et al., 2016)
**2016**	T, Q	8–16	174	FR	10.1111/cpf.12202 (Vanhelst et al., 2016)
**2015**	T, A, Q, B	9–19	1,467	US	10.1371/journal.pone.0138175 (Barnett et al., 2015)
**2015**	T, A, B	12–19	410	HU	10.1080/02701367.2015.1042800 (Laurson et al., 2015)
**2015**	T, A, Q, B	10–18	2,602	HU	10.1080/02701367.2015.1043231 (Welk et al., 2015)
**2015**	T	13–14	324	UA	10.7752/Jpes.2017.S5237 (Galan et al., 2017)
**2015**	T, A, Q	18–28	300	US m	10.1249/MSS.0000000000000396 (Crowley et al., 2015)
**2013**	Q, M	16–19	659	FI	10.1186/s12889-016-3880-5 (Hankonen et al., 2017)
**2012**	T, A	4–80	31,349	FR	10.1136/bmjopen-2012-001022 (Nassif et al., 2012)
**2008**	T, Q	13–18	71	NO	10.1186/1471-2288-8-47 (Rangul et al., 2008)
**2007**	A	19–26	140	FI m	10.1249/mss.0b013e318155a813 (Mattila et al., 2007)
**2006**	T, Q	11–16	94	BR	10.1590/s0034-89102006000600009 (Florindo et al., 2006)

a ISO country codes (Alpha-2).

b E6 = GR, DE, FR, HU, AT, ES; E7 = DE, GR, IE, NL, PL, ES, GB. The identification of methods is identical to that used in Figure 3 (A, anthropometry; B, biochemical analysis; C, cardiorespiratory fitness; M, accelerometer; Q, questionnaire; T, PA test).

Thus, data obtained from a questionnaire may be distorted due to subjective assessment. For this reason it seems advantageous to combine a questionnaire with physical testing, even though the correlation between the perceived and actual level of PA is significant (Štefan et al., 2019).

The physical fitness of adolescents is assessed by means of various test batteries (Marques et al., 2021). In this articles included in the search, FITNESSGRAM is the most common (Clark et al., 2015; Laurson et al., 2015; Welk et al., 2015; Davis et al., 2016; Cadenas-Sanchez et al., 2017; Júdice et al., 2017; Agostinis-Sobrinho et al., 2018; Chen et al., 2018). The specific components of the FITNESSGRAM^®^ are curl-ups (abdominal strength and endurance), push-ups (upper body strength and endurance), sit and reach (flexibility of the hamstrings and the lower back), the Progressive Aerobic Cardiovascular Endurance Run (PACER) test (CRF), and BMI (measure indicating the appropriateness of a young person's height/weight ratio) (Júdice et al., 2017). Other test batteries used were e.g., EUROFIT (Tishukaj et al., 2017; Corredor-Corredor et al., 2019; Štefan et al., 2019), the ALPHA-FIT test battery (Fernández et al., 2017; Chrismas et al., 2019), and the BOUGE physical fitness battery (Vanhelst et al., 2016). The variation of these test batteries results in difficulties when comparing them. Methods do exist, nonetheless, that make it possible to mutually compare batteries (they are based on *z*-scores), but this topic will be addressed in another article.

The overall characteristics of studies are summarized in Table 1. It shows the year of the article's publication, the methods used for assessing fitness or PA (see the legend to Figure 3), the age and number of respondents included in the study, the country of the research, and the digital object identifier (DOI) of the journal article.

### Individual factors in relation to PA

Longitudinal studies that would make it possible to identify changes of movement behavior in the course of adolescence and analyse the effect of such changes on the health of an individual are not available. Studies typically examine the relationship between PA and an individual's health in terms of both the psychological and physical aspects of the individual, in particular in connection with obesity (Köchli et al., 2019; Ruedl et al., 2019), with BMI (Bi et al., 2019), with cardiometabolic risk (Roldão da Silva et al., 2020), or with adherence to a specific diet (Galan-Lopez et al., 2019), but without any long-term monitoring of the tested individuals.

Vigorous PA as well as greater and increasing muscular fitness in youth are associated with lower levels of blood pressure across the adolescence (Agostinis-Sobrinho et al., 2018). Vigorous PA, good cardiorespiratory fitness and a healthy weight (BMI) are associated with mental wellbeing and quality of life (Lang et al., 2019). Percentage of fat is ranked as the most important correlate for Total, Physical and Psychosocial HRQoL (Health-Related Quality of Life) (Tsiros et al., 2017; Eddolls et al., 2018).

Many studies assess the role of physical fitness on mental wellbeing and health or, conversely, the role of mental health on physical performance (Fossati et al., 2021). The positive relationship between physical activity and mental health is well established, and the COVID-19 pandemic has increased people's attention to the positive effects of physical activity (Fossati et al., 2021; Nie et al., 2021; Lou et al., 2023).

Physical activity releases endorphins, the so-called “happiness hormones,” which can improve mood and reduce feelings of anxiety and stress. Achieving physical goals and improving fitness can lead to increased self-esteem and a sense of achievement. This positive effect on self-esteem can also carry over into other areas of life. Regular physical activity can also help improve sleep quality (Dolezal et al., 2017; Wang and Boros, 2021).

Physical exercise (PE) induces structural and functional changes in the brain, which that has huge benefits for cognitive function and wellbeing. Physical activity is also a protective factor against neurodegeneration (Mandolesi et al., 2018). Studies show that regular exercise can improve memory, concentration and decision-making (Mandolesi et al., 2018; Marin Bosch et al., 2021).

There are also studies which support an association between low physical fitness and depressive symptoms. There were 300 soldiers included into the study. Depressive symptoms were 60% lower for soldiers in the high fitness category (odds ratio: 0.40; 95% confidence interval: 0.19–0.84) compared with soldiers in the low fitness category (Crowley et al., 2015).

Physical activity declines with age. For example, according to a Norwegian study (Lagestad et al., 2018), at the age of 14, 61% of boys were classified as active, while at the age of 19, only 11% were physically active. Furthermore, body mass index increased during the period for both genders, while oxygen uptake decreased. Similar results are also shown by one study (Niessner et al., 2020) which examined the development of physical fitness in German youth and provided its percentile curves, and another study (Weston et al., 2019) which assessed, as part of a preliminary study, the PA of younger school children. The attitude to PA also changes with age—see the study (Kuśnierz et al., 2020) which shows that younger children prioritize the “fun” aspects of PA whereas for older children “fitness” is more important. Therefore, it is important to develop good habits and a healthy lifestyle in childhood. This increases the chance that they will be maintained into adulthood.

It turns out that PA is influenced by the environment in which we live, the options we have and are able to utilize. Increased opportunities for fitness and recreation were significantly associated with increased adolescent PA (Nicosia and Datar, 2018). Similar conclusions were also derived in one study (Arena et al., 2019) that examined health behaviors to fitness measures among boy scouts, which showed a clear difference between average adolescents and those who were members of a scout association.

Schools seem to be an ideal environment for supporting PA. However, education systems differ and the requirements placed on students as well as the availability of organized activities vary. There are hardly any studies that compared education systems (Bauman et al., 2011; Lee and Choi, 2011). For students it is important to participate in organized PAs, or to be a member of a sports club. Such participation has a positive effect not only on people's physical attributes, but also on their social and psychological situation. The broader the range of organized activities offered, the better the PA of students (Zullig and White, 2011). The recommendations for vigorous PA are met by 45.9% of Czech boys and 33.4% of girls; and by 64.5% of Polish boys and 51.3% of girls. Participation in organized PA was the main correlate to achieving recommendations for vigorous PA (Frömel et al., 2018).

Another important factor is gender. Studies suggest that boys are more competitive in sports than girls and participate in extracurricular activities more often (Chrismas et al., 2019). Boys had significantly higher actual and perceived object control competence (ball skills), moderate-to-vigorous PA (MVPA), cardiorespiratory fitness, and upper and lower musculoskeletal fitness than girls (Barnett et al., 2015, 2018; Clark et al., 2015). The decrease in girls' PA could be linked to the fact that during their adolescent years a high percentage of girls do not like PE classes. It is therefore advisable to create PA programmes that are also attractive for girls, and that make it possible to use flexibility, which is one of the disciplines in which girls are better than boys (Lee et al., 2017). Thus, it is useful to ascertain which sports bring the greatest joy to adolescents. According to one study (Wilkinson and Bretzing, 2011), girls preferred fitness units to sports units. As for fitness activities, the activities most popular with girls include pilates, aerobics, step aerobics, and kickboxing (Wilkinson and Bretzing, 2011). These are good lifetime activities that students can easily do on their own at home, rather than having to find a team to play the sport with or attend a specific facility.

Sports performance or movement activities of children in general are also considerably influenced by motivation from their parents (Wiersma, 2001) and coaches (Atkins et al., 2015). Studies (García Bengoechea et al., 2017; Oliveira-Brochado et al., 2017) show that the mapping the psychological background of respondents could be important in relation to motivation for doing sports.

There are hardly any studies examining the topic in a manner that might coincide with our particular interest, i.e., studies connecting PA and (pre)military service. Where such studies exist, they focus on a selected specific characteristic or circumstances relating to the army. An early study from 2007 (Mattila et al., 2007) refers to the fact that existing physical training programmes are not able to provide the optimal level of functional readiness required by military students and officers attending military universities to perform professional tasks. The solution to this problem, according to scientists, is to successfully adapt modern techniques of intensive functional training (crossfit) into the physical training process of cadets and military students. All the studies we managed to find (Table 1) agree that PA and healthy behavior have been on the decline in recent years. This decline usually involves changes in the level of PA in relation to other factors, such as the body composition of healthy male Finnish conscripts (Jurvelin et al., 2020), eating patterns, alcohol consumption, cigarette smoking, and e.g., psychological distress in Lithuanian military conscripts (Mieziene et al., 2020); other factors include BMI, weight status, and socioeconomic status in Swedish conscripts (Lissner et al., 2019). Elsewhere (Jurvelin et al., 2020), PA is studied in connection to the training load during basic training experienced by Finnish conscripts, where it is recommended during military service to customize the physical training of conscripts as much as possible in relation to their fitness levels. The most recent study (Pearlman et al., 2019) focuses on the families of military dependents, namely on adolescents aged 12–17; however, the study assesses so-called weight-based teasing with the factors: BMI, eating disorders, and psychological effects.

The systematic review is not without its limitations. The terms selected to identify the relationship between PA and military readiness, although very thorough, may have excluded documents that did not meet the criteria set. The search was carried out in only four databases, with the addition of publications archived in the library. It is also possible that some studies are missing, which may slightly bias the review; it is not clear whether such studies are unpublished or were published in local languages and therefore could not be retrieved. Nevertheless, the main contribution of this review is the number of articles reviewed to describe the methods of measuring and individual factors influencing the PA in adolescents and to identify the gap in the topic of our interest, i.e., the studies connecting PA and (pre)military service.

## Conclusion

The field of PA and fitness in adolescents is addressed by a broad community of experts, but there are no publications concerning PA and (pre)military service. There are also no longitudinal studies. The search authors specially focused on this type of publication, but despite their efforts they did not find any other publications than those listed in Table 1. In addition to the list of articles in Table 1, the search includes a list of methods that are currently used to assess physical fitness. The method most commonly used in the examined articles was the combination of a PA test and anthropometry. Combing these two methods with a questionnaire was also common. Among other things, the search also dealt with factors that may influence movement activity and factors influenced by movement activity.

PA in adolescents is one of the important factors influencing the health and life of adolescents and, consequently, the army of the given country. The Czech Army recruits new members from among the adult citizens of the Czech Republic with a clean criminal record and appropriate education, capabilities, age, and health. Training after the commencement of service requires the use of methods and means that are appropriate for the level of new recruits and that will secure the minimum required level of overall preparedness. Motivated, capable, well-trained and qualified military personnel are necessary for the success of any army. Therefore, the planning and management of training requires information about the condition of selected target groups of citizens of the Czech Republic, so that the training system is prepared for the condition of future participants and where appropriate supplemented with new features. The results ascertained in this publication will be used in the project BODY—Physical Fitness of the Population as a Risk Factor for Ensuring the Defensive Capacity of the Czech Republic. In this project the following tools will be used (as in the articles above): questionnaire survey (Q), biochemical analysis (B), anthropometry (A), a PA test (T), and cardiorespiratory fitness (C). We found only one study of this extent and type (International Coordinating Centre, 2021). We will supplement the aforesaid methods with psychological questioning (questionnaire NEO-PI-R).

The project BODY began in 2020 and will be investigated until the end of 2023. In 2020, partly owing to the negative development of the COVID-19 pandemic, only the first “preparatory” stage of the project was carried out. The first major tangible results will be achieved after the comprehensive monitoring of target groups of students attending secondary and vocational schools (and elementary schools, if appropriate) within the following catchment areas: nationwide, rural, urban, and city. The required data will be gathered in a manner ensuring that the obtained sample characterizes the target population with sufficient precision. In each of these target groups we will monitor, on an individual basis, the condition of the body, musculoskeletal system, selected movement abilities and skills, the change dynamics of selected blood analytes, and mental health once a year. The data will be statistically processed and, on the basis of their 3-year course, we will approximate their possible development.

The project will include an analysis of the degree of divergence between the approximate and desired situation with regard to the expected needs not only of the Czech Army, but an entire society currently affected by the post-COVID syndrome. Based on the information gathered, we will propose modifications to the current teaching of PE at secondary schools (and basic schools, if appropriate), together with recommendations for changes to the content of preparatory courses run prior to testing the fitness of potential recruits to the Czech Army.

## Data availability statement

The original contributions presented in the study are included in the article/supplementary material, further inquiries can be directed to the corresponding author.

## Author contributions

All authors listed have made a substantial, direct, and intellectual contribution to the work and approved it for publication.
